# Novel detection of mutation in the *TECPR2* gene in a Chinese hereditary spastic paraplegia 49 patient: a case report

**DOI:** 10.1186/s12883-022-02572-x

**Published:** 2022-02-07

**Authors:** Yalin Guan, Hui Lu, Wenchao Zuo, Xiaodan Wang, Shimin Wang, Xinping Wang, Feng Liu, Kun Jia, Rui Gao, Hao Wu, Zhihong Shi, Yong Ji

**Affiliations:** 1 Department of Neurology, Tianjin Huanhu Hospital, Tianjin, 300350 China; 2 Tianjin Key Laboratory of Cerebral Vascular and Neurodegenerative Disease, Tianjin, 300350 China

**Keywords:** Hereditary spastic paraplegia, Autosomal recessive inherited disease, Spastic ataxia, Heterozygous mutations, Case report

## Abstract

**Background:**

Hereditary spastic paraplegia 49 (HSP49) is an autosomal recessive genetic disease first discovered in 2012; and which the mutation primarily affects Bukharian Jewish patients.

**Case presentation:**

The present case reports the first instance of HSP49 detected in China. The patient had normal mental development and good athletic ability before 10 years old and presented with instable temperature, mental retardation, spastic ataxia, and paroxysmal convulsions. Genetic diagnosis was based on detection of whole exons and two heterozygous variants in the exon region of the *TECPR2* gene: c.1729C > T and c.4189G > A. Mutations at these two sites have not been previously reported.

**Conclusions:**

This case expands the gene mutation spectrum and clinical phenotypic characteristics of autosomal recessive HSP in China; moreover, it indicates differences in the clinical phenotype of HSP49 in different ethnicities. In addition, this reported provides further evidence regarding the effectiveness of targeted next-generation sequencing technology in improving the efficiency and diagnostic rate of genetic diagnosis of HSP.

## Background

Hereditary spastic paraplegia (HSP) is a group of familial diseases characterized by progressive degeneration of the corticospinal tract. The emergence of next-generation sequencing has allowed recognition of the extreme variability of genes and mutations in HSP [1, 2]. The inheritance mode of HSP includes autosomal dominant inheritance, autosomal recessive inheritance, and X-linked inheritance [1]. Its gene locus is termed spastic paraplegia [3], and it is numbered sequentially. To date, the number of discovered pathogenic gene loci exceeds 83 and continues to increase [4].

Hereditary spastic paraplegia 49 (HSP49) was first reported by Oz-Levi et al. in 2012 [5] and is an autosomal recessive inherited disease caused by mutations in the *TECPR2* gene. HSP49 mainly manifests as recessive inherited psychomotor retardation, mental retardation, spastic ataxia, and recurrent apnea. Previous reports indicated that HSP49 was due primarily to homozygous mutations in a Bukharian Jewish population [5] and heterozygous mutations in non-Bukharian Jewish populations [6, 7]. Most HSP49 cases have been reported in Israel, while no cases have been officially reported in China. This article reports a case of a Chinese patient with HSP49 caused by a heterozygous gene mutation.

## Case presentation

The patient in the present case was a 19-year-old male. His parents are of Chinese descent and are healthy and not consanguineous. They were not reported in his extended family. He was born via cesarean section at 39 weeks of gestation in March 2001 with a birthweight of 3200 g. Before 10 years of age, the patient’s intelligence and athletic ability were unaffected. He had excellent performance in an English speech contest and in swimming.

In February 2011, at the age of 10 years old, a high fever persisted for 4 days, after which an epileptic seizure occurred several times in one day; one day later, the patient exhibited a persistent loss of consciousness and dyspnea. Following treatment with antiepileptic drugs, glucocorticoid, plasma exchange, and endotracheal intubation, the patient’s symptoms were controlled. However, the patient exhibited the following sequelae: severe cognitive impairment, personality change, and aphasia.

In the following 9 years, the patient experienced frequent recurrent seizures. Moreover, he presented a paroxysmal temperature increase 1–2 times daily during which body temperature suddenly increased from 36℃ to higher than 38℃ without infective symptoms and which self-remitted after a few hours. The walking ability of the patient gradually decreased, and in 2018, he needed wheelchair or walking with assistance from his parents. He had normal digestive function without acid reflux heartburn symptoms; he rarely had pulmonary infection. The patient slept normally at night and had no abnormal breathing during sleep.

Three months before admission, on February 2020, his caregiver forced him to take prescribed medicine because he refused to take the medicine orally; this led to a repeated cough, choking, dysphagia, aspiration pneumonia, and an increase in limb seizures, which occurred about 10 times per day, each lasting 1–10 min. At admission, the patient was in a moderate coma. Pupils were round and reactive to light. The neck was supple with immobility, and there was low muscle tension of the four limbs with positive and symmetric deep-tendon reflexes, which is a negative bilateral pathological sign. Lung auscultation revealed shortness of breath and extensive rales. Cardiac examination revealed normal heart sounds without murmurs and appropriate peripheral perfusion. The abdomen was soft without organomegaly.

Laboratory tests showed no obvious abnormalities in routine blood, urine, and stool tests. Autoantibodies for autoimmune encephalopathy and demyelinating diseases of the central nervous system, such as myelin oligodendrocyte glycoprotein, aquaporin-4, and myelin basic protein, were all negative. Syphilis, AIDS, and hepatitis were all negative. Lumbar puncture showed that cerebrospinal fluid pressure was normal with white blood cells 2 × 10^6^/L, protein 0.65 g/L, and lactate 2.6 mmol/L; all cerebrospinal fluid cultures were negative.

**Fig. 1:**
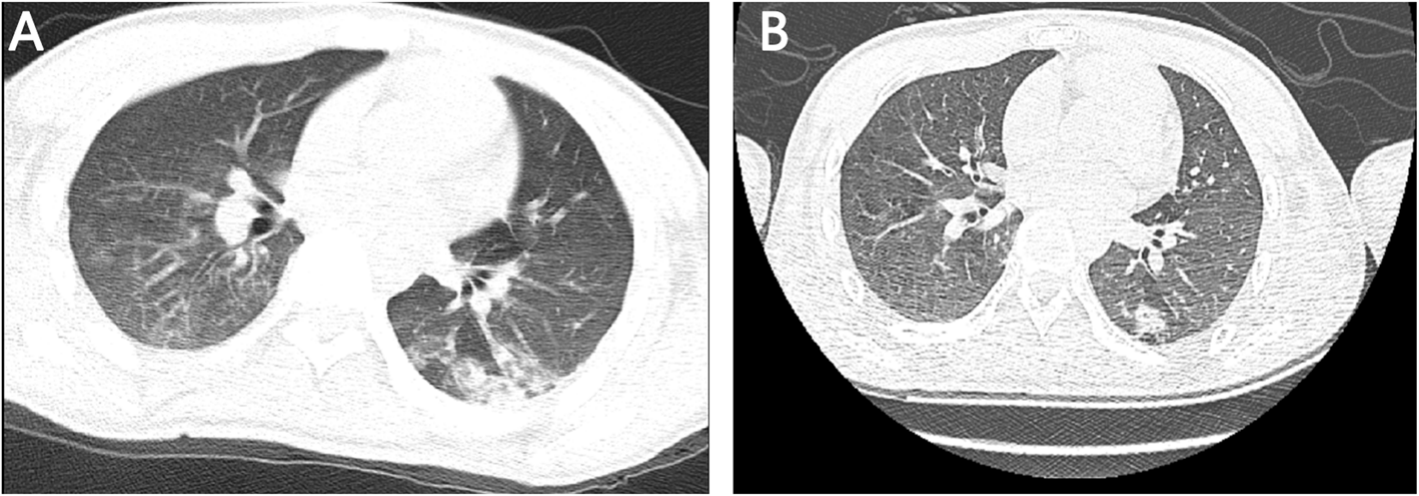
Chest CT findings. After admission, the patient’s chest CT showed bilateral pulmonary infection, especially in the inferior lobe **(A)**. Eight days after admission, chest CT reexamination showed that bilateral pulmonary infection was controlled **(B)**

**Fig. 2:**
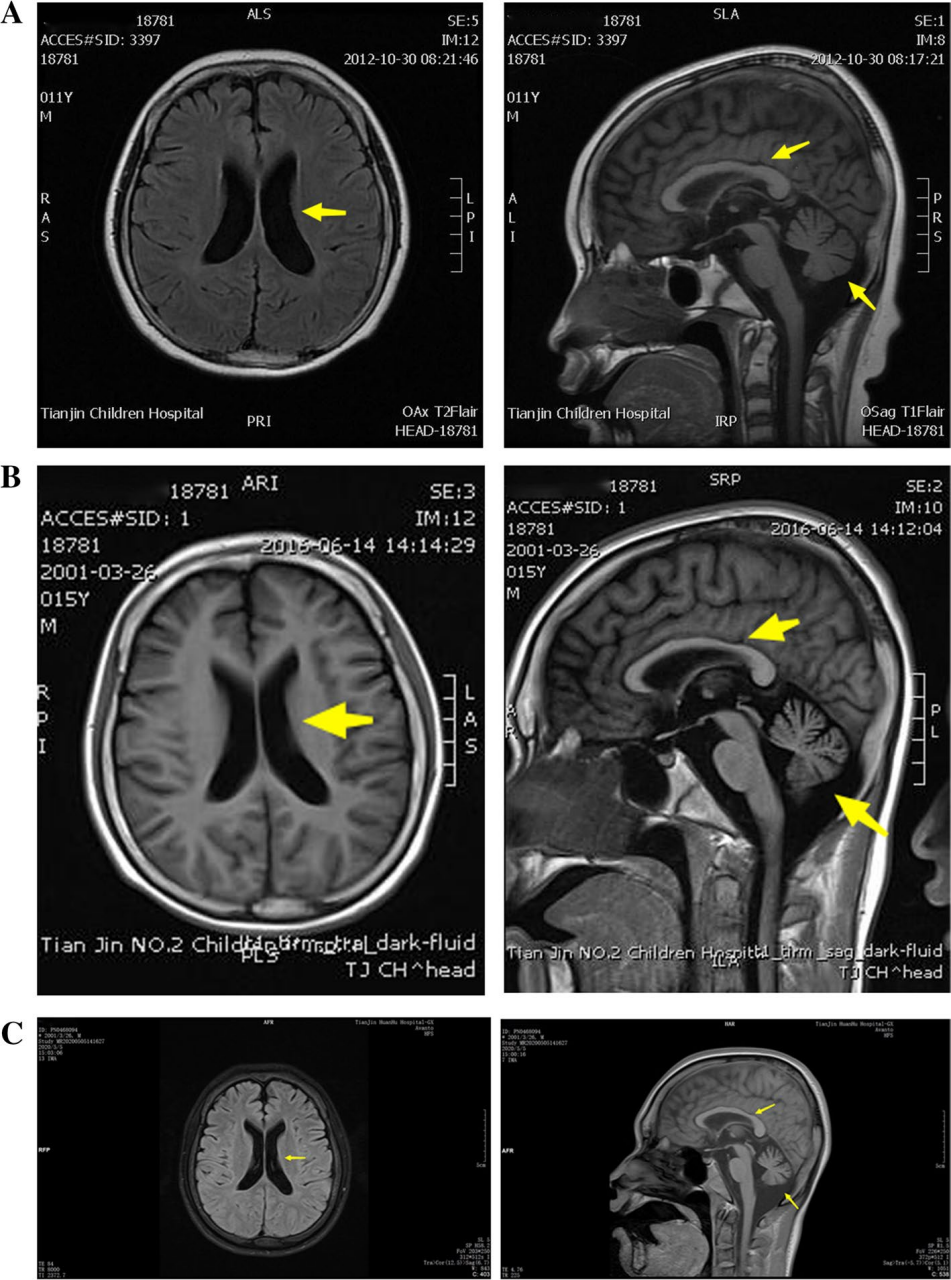
Brain MRI findings. the brain MRI showed mild enlargement of lateral ventricle, corpus callosum was normal, and partial widening of the cerebellar sulci in 2012 **(A)**; enlargement of lateral ventricle, partial thinning of corpus callosum, significant enlargement of the cerebellar sulci in 2016 **(B)**; Cerebellar atrophy and thinning of corpus callosum were more serious than before in 2020 **(C)**

**Fig. 3:**
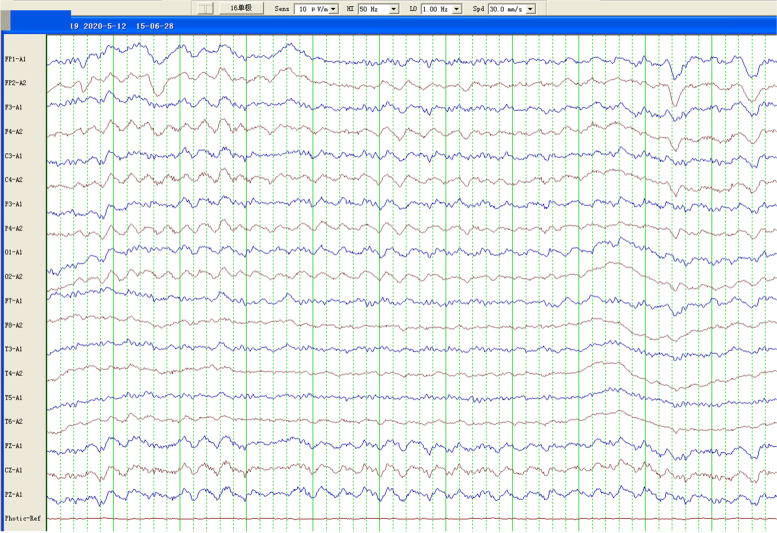
Brain EEG findings. During the awake period, a large number of bilateral symmetric 3-4c/s low and medium amplitude irregular slow waves were found, mainly in the anterior head and without obvious epileptiform waves

On admission, chest CT showed bilateral pulmonary infection, especially in the inferior lobe (Fig. 1A). The brain magnetic resonance image (MRI) of the patient after admission is shown in Fig. 2C. Moreover, compared with MRIs conducted in 2012 (Fig. 2A) and 2016 (Fig. 2B), the patient had a trend in which the cerebellar sulci significantly widened along with severe cerebellar atrophy, thinning of the corpus callosum, and mild enlargement of lateral ventricle. Moreover, an electromyogram showed that normal nerve conduction velocity, and skin sympathetic reaction suggested autonomic neuropathy. An electroencephalogram after admission showed that during the awake period, a large number of bilateral symmetric 3-4c/s low and medium amplitude irregular slow waves were found, mainly in the anterior head and without obvious epileptiform waves (Fig. 3).

**Fig. 4:**
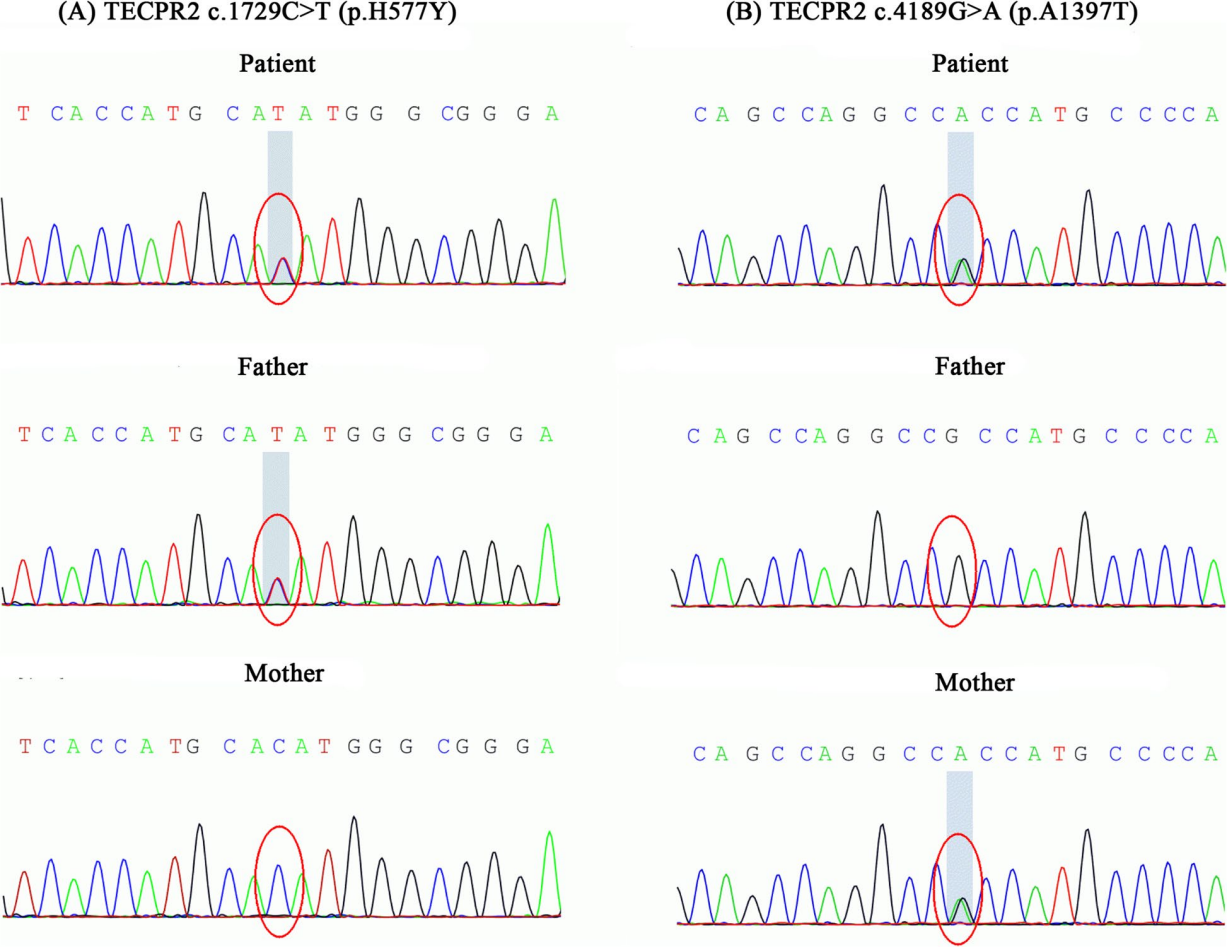
Missense variants in the patient, his mother, and his father by WES. **A** variant c.1729C > T caused the amino acid change p.H577Y in TECPR2 and was inherited from the father. **B** variant c.4189G > A caused the amino acid change p.A1397T in TECPR2 and was inherited from the mother

Whole-exome sequencing (WES) was employed to obtain a potential genetic diagnosis after admission. WES revealed compound heterozygous variants in the *TECPR2* gene. Both variants were missense mutations. The first mutation was c.1729C > T, causing an amino acid change of p.H577Y (NM_014844.3) in the *TECPR2* protein. The second missense variant was c.4189G > A, causing the amino acid change p.A1397T (NM_014844.3). Neither mutation has been previously reported in patients with HSP49, and they are not currently in the HGMDpro database [8]. The mutation c.1729C > T (p.H577Y) was inherited from the father, and c.4189G > A (p.A1397T) was inherited from the mother, which ultimately made the patient a compound heterozygote for these variants (Fig. 4).

After admission, the patient’s oxygen saturation decreased intermittently, reaching a nadir of 70%. The patient was treated with sputum suction and other airway nursing and cephalosporin antibiotics. After treatment, the infection was gradually controlled, consciousness turned clear, and blood oxygen saturation returned to normal. Eight days after admission, chest CT reexamination showed that bilateral pulmonary infection was controlled and had improved (Fig. 1B).

During hospitalization, the patient's temperature increased paroxysmally, peaking at 38℃. Routine blood work and procalcitonin levels were normal and the blood culture was negative, yet the patient's temperature still increased intermittently. At this time, the patient had no symptoms of infection and showed similar temperature fluctuations as those over the previous years before admission, which indicated possible autonomic dysregulation. The patient's digestive function was good after mental recovery, and gastroesophageal reflux disease was excluded after gastroenterology consultation. Sleep apnea syndrome was also excluded after sleep monitoring.

Moreover, seizures occurred recurrently after admission. Lamotrigine was provided to control seizures, and the onset frequency decreased until there was no epilepsy occurrence for up to 17 days. The patient was also provided idebenone to protect mitochondria, tizanidine to reduce muscle tension, and other nutritional nerve treatments.

The patient was discharged at 30 days of hospitalization. Discharge diagnosis was HSP49, secondary epilepsy, and pulmonary infection. At the time of discharge, the patient was conscious, aphasic, still had severe cognitive impairment and ataxic gait, and required walking assistance. Both upper limb tendon reflexes were +  + , left lower limb tendon reflex was + , right lower limb tendon reflexes were +  +  + , and bilateral pathological signs were not elicited. Follow-up was conducted 3 months later. The patient was prescribed lamotrigine and levetiracetam to control epilepsy, but he still had intermittent seizures, approximately once weekly.

## Discussion and conclusion

HSP49 was first reported by Oz-Levi et al. in 2012 [5] as an autosomal recessive genetic disease mainly caused by *TECPR2* mutations. *TECPR2* is a binding partner of the mammalian Atg8 protein family and a possible positive regulator of autophagy [9–11]. Fraiberg et al. [12] showed that the protein encoded by *TECPR2* is involved in the targeting of autophagosomes to lysosomes; this process is impaired by mutations in the *TECPR2* gene, resulting in autophagosomes that cannot be eliminated and for which the accumulation leads to neurodegenerative diseases. To improve understanding of *TECPR2* gene function, Tamim-Yecheskel et al. established a *TECPR2* knockout mouse model. *TECPR2* -/- mice displayed clinical manifestations similar to those of HSP49 patients, including age-dependent neurodegenerative diseases. Moreover, abnormal accumulation of autophagosomes occurs in *TECPR2* -/- mice in an age-dependent manner [13]. HSP49 displays clinical heterogeneity, which may be related to the diversity of *TECPR2* gene mutation. Patients with HSP49 that harbor homozygous mutations exhibit obvious deformities, developmental delays, and hypotonia; these features typically manifest as mental retardation, spastic paraplegia, dysarthria, and ataxia. Characteristic dysmorphic features include microcephaly, dental crowding, a short broad neck, and a chubby appearance [5, 14].

Another clinical report regarding three children with mutations in *TECRP2* also described outstanding features of sensory autonomic neuropathy that manifested as early hypotonia; areflexia; decreased pain and temperature sensitivity; and unstable blood pressure, body temperature, and osmotic pressure. The author proposed that the disease should therefore be classified as a new subtype of hereditary sensory-autonomic neuropathy, because in addition to intellectual disability and evolving spasticity, the main disabling feature of this unique disorder is autonomic-sensory neuropathy [6]. In contrast, clinical manifestations with heterozygous mutations are relatively mild, such as an Italian girl with HSP49 who displayed no impairment in mental development [7].

The patient in the present case also harbored heterozygous mutations and exhibited milder clinical symptoms than those in patients with homozygous mutations. The present patient had normal mental development and good athletic ability before the age of 10 years. Although the patient showed central hypoventilation during lung infection, there was no dyspnea or hypoventilation after the lung infection was resolved. At present, the patient only presented with mental retardation, spastic ataxia, and paroxysmal convulsions. Moreover, the patient has paroxysmally high body temperature even in the absence of infection, indicating possible signs of autonomic dysregulation. The MRI of this patient is consistent with previous reports: severe cerebellar atrophy, enlargement of the fourth ventricle, thinning of the corpus callosum, and mild enlargement of lateral ventricle. Finally, the diagnosis of this patient was based on the detection of whole exons and two heterozygous variants in the exon region of the *TECPR2* gene: c.1729C > T and c.4189G > A. However, according to the HGMD pro database search, these two variant sites have not been previously reported. Although the pathogenicity of these two new mutations has not been determined, previous literature on the pathogenicity of other mutations in the *TECPR2* gene suggests [5, 7, 14] that these two mutations are likely to be responsible for the *TECPR2* gene abnormality and the present phenotype of the patients. At present, we only report the clinical manifestation and heterozygous variants in the *TECPR2* gene of this patient. Future studies will further explore the pathogenicity of mutation of c.1729C > T and c.4189G > A in animal experiments.

The mild clinical manifestations of the present patient may be because the mutation site of the *TECPR2* gene differs from those previously reported. However, as illustrated in animal models, the accumulation of autophagosomes caused by the abnormality of the *TECPR2* is age-dependent; therefore, we will diligently follow up the patient's subsequent development, with particular attention to changes in patient's intelligence and muscle strength so that we can enact timely treatment.

The current case expands the gene mutation spectrum and clinical phenotypic characteristics of autosomal recessive HSP in China. In addition, this case shows that there are certain differences in the clinical manifestations of HSP49 in different ethnic groups. Moreover, it provides further evidence regarding the effectiveness of targeted next-generation sequencing technology in improving the efficiency and diagnostic rate of genetic diagnoses of HSP.

## Data Availability

The datasets used and/or analysed during the current study are available from the corresponding author on reasonable request.

## Abbreviations

HSP49: Hereditary spastic paraplegia 49
HSP: Hereditary spastic paraplegia
MRI: Magnetic resonance image
WES: Whole-exome sequencing
